# Framing Messages to Deal With the COVID-19 Crisis: The Role of Loss/Gain Frames and Content

**DOI:** 10.3389/fpsyg.2021.568212

**Published:** 2021-01-28

**Authors:** Carlos Gantiva, William Jiménez-Leal, Joan Urriago-Rayo

**Affiliations:** ^1^Psychology Department, Universidad de los Andes, Bogotá, Colombia; ^2^Laboratorio de Cognición, Universidad de los Andes, Bogotá, Colombia

**Keywords:** framing effects, health communication, COVID-19, risk perception, behavioral science

## Abstract

The goal of this study was to test the role of message framing for effective communication of self-care behaviors in the context of the COVID-19 pandemic, contrasting health and economic-focused messages. We presented 319 participants with an unforced choice task where they had to select the message that they believed was more effective to increase intentions toward self-care behaviors, motivate self-care behaviors in others, increase perceived risk and enhance perceived message strength. Results showed that gain-frame health messages increased intention to adopt self-care behaviors and were judged to be stronger. Loss-framed health messages increased risk perception. When judging effectiveness for others, participants believed other people would be more sensitive to messages with an economic focus. These results can be used by governments to guide communication for the prevention of COVID-19 contagion in the media and social networks, where time and space for communicating information are limited.

## Introduction

The pandemic caused by the novel coronavirus COVID-19 (World Health Organization [WHO], 2020a) during 2020, has resulted in hundreds of thousands of deaths around the world, and has consequently led governments to take extraordinary measures to face it. The development and commercialization of a vaccine for COVID-19 will be a long and expensive process. The cost of developing a vaccine for an infectious disease is estimated to be between 1.2 and 8.4 billion dollars (Gouglas et al., 2018), and the process to produce a licensed vaccine typically takes many years (Lurie et al., 2020). Therefore behavioral change and modification (i.e., hand washing, physical distance and staying home) is one of the main strategies to manage the pandemic (World Health Organization [WHO], 2020b). Governments have encouraged the population to adopt these behaviors through messages in mass media and social networks, hoping people would develop new habits and in doing so help reduce or postpone the number of contagions and the strain on health systems.

Behavioral economics has shown that using gain-loss frames to communicate information impacts decision making, risk perception, and behavioral intention (Kahneman, 2003). In the context of health communication, gain-framed messages emphasize the benefits or the positive outcomes that are accrued through adopting the behavior. On the other hand, loss-framed messages attempt to persuade by pointing at the negative consequences or costs incurred by not adopting the recommended behavior (Rothman and Salovey, 1997). Research in this field has found that, for example, gain-framed messages are more effective in motivating healthy eating behaviors (Roberto and Kawachi, 2014), while loss-framed messages are more effective to motivate breast self-examination (Williams et al., 2001) and to quit smoking (Nan et al., 2015).

Recently, several systematic and theoretical reviews have been conducted to propose how behavioral sciences could contribute to managing the COVID-19 outbreak (Lunn et al., 2020a; Van Bavel et al., 2020). However, the empirical evidence is scarce. Lunn et al. (2020b) used negative-framed messages to effectively motivate social distancing in Ireland. However, this study aimed to identify effects of communication strategies already implemented. So far there have been no studies to systematically identify the characteristics and structure that messages should have in order to effectively motivate population to change or adopt new behaviors such as frequent hand washing and physical distancing.

It is also plausible that cultural variation can play a role in the impact of these messages. We foresee two dimensions along which cultural differences could emerge. First, focusing on a particular content and/or frame might be more or less effective depending on particular countries and communities. Many citizens and national governments around the world have expressed concerns about the economic impact of public health measures implemented (McKee and Stuckler, 2020). Some even ponder whether the public health measures centered around stringent lockdowns could result in even worse consequences due to the psychological and economic consequences of unemployment, bankruptcies and social isolation (Singer and Plant, 2020; Tankersley, 2020). An open question is whether the public is also sensitive to these concerns and whether its effect would interact with a loss/gain frame. This question is especially relevant for societies where economies are more fragile and thus their citizens are more likely to be more responsive to economic concerns.

Second, personal and injunctive norms vary greatly between societies. Injunctive norms refer to perceptions of what others approve or encourage and have been shown to be closely related to personal intentions (Ball et al., 2010; Smith et al., 2012). There is evidence that misperception or underestimation of these social norms has an impact on engaging in behaviors falsely believed to be common or accepted in a group or community. Research and interventions based on social norms has mostly been done on alcohol and substance abuse related behaviors (McAlaney et al., 2011), but we believe it offers an interesting tool in the context of COVID-19. By identifying and assessing the gap between what one believes would be a good message for oneself versus others in one’s context, not only makes it possible to map out the extent of the misperception but also to intervene to correct it (Dempsey et al., 2018).

The objective of the present study was thus to evaluate the impact of gain-loss frames and the content of the message (health/economy) on self-reported motivation to engage in self-care behaviors (i.e., hand washing, physical distance, and staying home), engage others in the same self-care behaviors, risk perception of contagion and perceived message strength. The results of this study will help policy makers to design more effective messages to mitigate the impact of COVID-19.

In line with this objective and previous research, we expected that gain-frames were more effective to motivate low-risk behaviors (i.e., hand washing) while loss-frames were more effective to motivate high-risk behaviors like staying at home, because for many this latter behavior jeopardizes employment. Thus, the effectiveness of the message depends both on the content (economic versus health) and the frame (low risk behaviors might be more effectively framed as gains while high cost behaviors could be better framed as losses). The preregistration, hypotheses, analysis plan, materials, raw data, and scripts for analysis are available online at the Open Science Framework^1^, in line with best practice in reproducible science.

## Materials and Methods

### Participants

A convenience sample of 319 subjects (69.9% female, 30.1% male), ranging from 18 to 60 years of age (*M* = 27.01, *SD* = 9.37) participated in the study. Participants were originally contacted through student’s university mailing lists from different faculties (e.g., social sciences, engineering, medicine, basic sciences, among others) and from one to final year, and through Facebook postings, where a short description of the study was included. Our sample comprised participants from the main cities in Colombia, all native Spanish speakers.

The sample was divided randomly into two groups: Frame message group (gain/loss) (*n* = 160) and Content message group (economy/health) (*n* = 159). Sample size was decided based on *a priori* power analysis for a crossed random effects design, assuming a power of *d* = 0.35 (*f* = 0.175) for a power of 0.95 to detect simple effects (and of 0.70 for a two way interaction) (see Supplementary Material). Table 1 summarizes the basic demographic characteristics of the sample.

**TABLE 1:** Demographic characteristics of the sample.

	Frame group (*n* = 160)	Content group (*n* = 159)	*χ^2^* or *t*	*p*
Age (years) [mean (SD)]	29.94 (9.34)	27.08 (9.44)	–0.13	0.89
Sex (% male)	33.1	27	1.40	0.23
Sex (% female)	66.9	73		
Educational level				
Medium (%)	45	45.3	0.003	0.95
High (%)	55	54.7		
Perceived socioeconomic status (range: 1–10) [mean (SD)]	5.98 (1.87)	5.98 (1.74)	0.001	1.00
Left–right political orientations (0: left, 100: right) [mean (SD)]	37.53 (22.29)	40.19 (22.61)	–1.05	0.29

*Educational level: Medium (participants who were currently in or finished high school or technology careers). High (participants who were currently in or finished university or postgraduate studies). SD, standard deviation.*

### Stimuli

We created eight messages related to consequences of following, or not, the self-care recommendations issued by public health authorities, so that half of these messages were gain-framed and the other half were loss-framed. In turn half the messages portrayed health consequences of the measures and the other half economic consequences. Each message was written in a white font on a black background to make them easy to read in either a mobile cellphone or a computer screen.

### Design and Procedure

The experiment used a 2 Frame (gain/loss) × 2 Content (health/economy) both-within-condition design (Westfall et al., 2014). Dependent variables were: (a) intentions of self-care behaviors, (b) perceived efficacy to motivate others to perform self-care behaviors, (c) perceived risk, and (d) perceived message strength (i.e., attention, importance, consequences expressions, and perceived effective to engage in self-care behaviors) (Nan et al., 2015). We decided to use this design because a fully factorial design would have likely led to effects of practice and fatigue (Bradley, 2009; Gantiva et al., 2019). The study was conducted between April 19th and 28th of 2020.

A Web-based experiment was conducted in Spanish on the *Qualtrics* platform. After digitally signing the consent form, participants were randomly assigned to one of the experimental conditions (*Frame* or *Content*). In both conditions participants had to choose one of the messages in an unforced choice task, for four pairs of messages. In the *Frame* condition, the two messages of each pair had the same content (either both on Health or both on Economics) while the frame (Gain/Loss) was systematically varied. On the *Content* condition, the two messages of each pair shared the same frame but varied in content (always Health vs. Economics). The display order of the stimuli pairs and the location (right or left) of each message were randomly determined.

**FIGURE 1:**
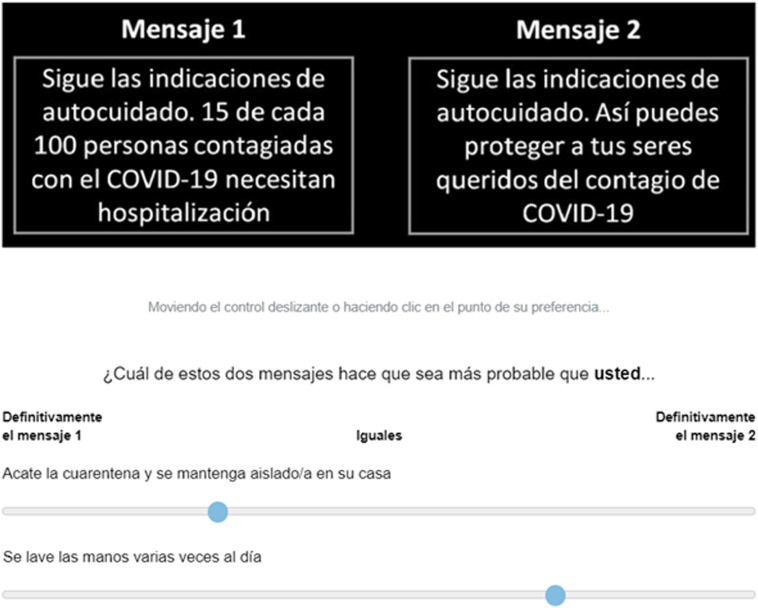
Example of message pairs in the *Frame* condition.

After seeing each pair of messages, participants had to choose the message of their preference for each dependent variable using a Visual Analog Scale (VAS) (see Figure 1). At the end of the experiment, participants responded to a set of questions on several demographic characteristics. The median duration time of the task was 10 min.

This study was carried out in accordance with the recommendations of the IRB of the University of los Andes (Approval #1169/2020), with written informed consent from all participants in accordance with the Declaration of Helsinki.

## Results

We derived a score per message by calculating the difference between the indifference point in the scale (50) and the final position of the slider selected by each participant for each pair. For example, when comparing two health messages, one with a gain-frame (left side of Figure 1) and the other with a loss-frame (right side of Figure 1); a participant might have selected 90, choosing the target on the right. In this case, this means that the loss-framed health message got assigned a score of 40 (i.e., subtracting the indifference point from the score, in this example 90 minus 50) while the gain-framed health message got a score of zero. When participants chose the indifference point, both messages got a score of zero. Manipulation checks showed that participants indeed recognized gain/loss-framed messages as such (over 91% of participants for all messages). Results below include the whole sample, since excluding data based on the manipulation check did not result in any difference.

We derived a perceived message strength index by averaging the scores assigned to the messages across the questions on attention, importance, consequences and perceived effective to engage in self-care behaviors (Nan et al., 2015). A reliability analysis showed very good internal consistency for these items (Cronbach’s α = 0.85, McDonald’s ω = 0.87), as expected.

**FIGURE 2:**
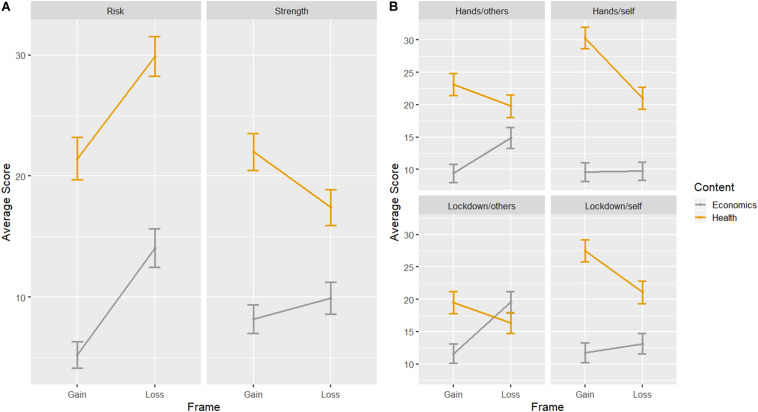
**(A)** Interaction between frame and content for risk communication (Risk) and perceived strength (Strength) of the message. **(B)** Interaction between frame and content for judged impact of hand washing on others (Hands/others) and on oneself (Hands/self) and judged impact of isolation measures on others (Lockdown/others) and oneself (Lockdown/self).

Analyses were conducted using the R statistical language (R Core Team, 2020). We fitted a series of linear mixed models with the *lme4* library (Bates et al., 2015) and performed corrected pairwise comparisons with the Tukey method with the *emmeans* library (Lenth, 2020), as per preregistration. However, all models resulted in singular fits, suggesting overcomplex model structures (Barr et al., 2013). Therefore, we fitted the same nested models as generalized linear models, omitting the random effect term for participants. Overall results are summarized in Table 2 and Figure 2.

**TABLE 2:** Summary of models fitted for each dimension.

	Risk	Strength	Hands/self	Hands/other	Lockdown/self	Lockdown/other
Health	26.00***	14.00***	22.00***	26.00***	20.00***	7.10***
	(23.0, 29.0)	(12.0, 16.0)	(20.0, 25.0)	(23.0, 30.0)	(17.0, 23.0)	(3.9, 10.0)
Loss	−0.71	1.70*	0.98	3.60**	2.90*	3.70**
	(−3.6, 2.2)	(−0.2, 3.7)	(−1.6, 3.6)	(0.4, 6.8)	(−0.2, 6.0)	(0.4, 7.0)
Frame condition	−0.56	−1.40**	8.60***	13.00***	6.10***	−0.63
	(−3.4, 2.3)	(−2.8, −0.04)	(6.0, 11.0)	(9.8, 16.0)	(3.0, 9.3)	(−3.9, 2.6)
Perceived social status		0.25				
		(−0.1, 0.6)				
Health: loss	3.80*	−6.30***	−1.40	−7.20***	−4.40*	−6.00**
	(−0.3, 7.8)	(−9.1, −3.6)	(−5.1, 2.3)	(−12.0, −2.7)	(−8.8, 0.05)	(−11.0, −1.4)
Health: frame condition	−19.00***		−17.00***	−21.00***	−12.00***	1.50
	(−23.0, −15.0)		(−20.0, −13.0)	(−26.0, −17.0)	(−17.0, −8.0)	(−3.1, 6.0)
Loss: frame condition	19.00***		1.50	−4.40*	5.20**	8.40***
	(15.0, 23.0)		(−2.2, 5.2)	(−8.8, 0.08)	(0.7, 9.6)	(3.8, 13.0)
Health: loss: frame condition	−8.20***		−9.70***	−1.40	−8.90***	−10.00***
	(−14.0, −2.4)		(−15.0, −4.5)	(−7.7, 4.9)	(−15.0, −2.6)	(−17.0, −3.6)
Constant	5.50***	7.40***	3.90***	5.30***	6.30***	12.00***
	(3.5, 7.6)	(4.7, 10.0)	(2.0, 5.7)	(3.0, 7.5)	(4.1, 8.6)	(9.6, 14.0)
N	2552	2552	2552	2552	2552	2552
Log likelihood	−11,079.00	−10,957.00	−10,825.00	−11,300.00	−11,289.00	−11,369.00
AIC	22175.00	21926.00	21665.00	22615.00	22594.00	22753.00

***p < 0.01; **p < 0.05; *p < 0.1. Number in brackets are the confidence intervals for each coefficient. Reference category is the economics content, gain frame, and content condition.

The best model for each dimension, except perceived message strength, includes simple and interactive effects for the main experimental variables: Content Type, Frame, and Experimental Condition. For perceived message strength, there was also an effect of perceived socio-economic status, so that people with higher status tended to perceive messages as stronger. However, this coefficient is not significant. Including other variables, such as gender and age, did not improve the models fit (e.g., including them did not result in greater variance explained in our dependent variables).

There are main effects of both *Content* and *Frame* type for all dimensions examined except when judging how good the messages were at convincing others to wash their hands. In all models fitted, gain-frames and Health-themed messages were always considered better [bearing in mind that interpretation of the main effects can be problematic in the presence of interactions (Salkind, 2010)].

We will now focus on the two-way interaction between *Content* and *Frame*, the main point of the study. There is an overall effect of *Content* for all dimensions so that health messages were considered more effective, better at communicating risk, and better to persuade oneself and others, except when assessing the impact of health messages on others regarding the lockdown. Messages focused on health and with a gain-frame were generally perceived as stronger than both loss-framed health messages (*M*_HealthGain_ = 22 vs. *M*_HealthLoss_ = 17.4, *z* = −4.90, *p* < 0.001) and gain-framed economics messages (*M*_EconomicsGain_ = 8.2, *z* = −12.80, *p* < 0.001), as can be seen in Figure 2A. On the other hand, loss-framed health messages were judged as better to communicate risk than any other type of message (all comparisons significant at *p* < 0.001).

The assessment of message impact on oneself and others is very similar for the two behaviors evaluated (hand washing and lockdown compliance). While in all cases the gain-framed health messages are considered more effective both for oneself and for others, the magnitude of the differences is smaller when judging the impact on others versus oneself. That is, people believed that others are more susceptible to economic-themed messages than they are. When considering the impact of messages on self-isolation measures (see Figure 2B), participants believe that other people are as receptive to gain-framed health messages as they would be to loss-framed economic messages (Figure 2B, lower right) while they considered themselves to be more influenced by gain-framed health messages and indifferent between loss and gain-framed economic messages (*z* = −1.30, *p* = 0.59). The pattern is similar for hand washing, but not as pronounced.

This pattern itself interacted with the experimental condition. This three-way interaction shows that the differences identified were stronger when the comparisons were between-content than when they were between-frames. That is, when people compared health versus economic messages, especially for gain-framed health messages, the differences tended to be larger than when the same message was paired with a loss-framed health message. This suggests an interesting joint evaluation effect, with potential real world repercussions (Hsee et al., 1999). We refrain from putting too much stock into this interpretation, since we did not make any predictions on this aspect of our design.

## Discussion

The aim of the present study was to evaluate the impact of message framing and content (health/economy) on several measures related to self-care behaviors (i.e., motivation to engage in self-care behaviors, engage others in the same self-care behaviors, risk perception of contagion and perceived message strength) in the context of the COVID-19 pandemic. The results showed that gain-framed messages were more effective to generate motivation to engage in self-care behaviors and were perceived as stronger. On the other hand, loss-framed messages were more effective at increasing awareness of risks. We also found that health messages were overwhelmingly preferred for all the measures, even though there is a tendency to judge that others may be more susceptible to economic messages than oneself. Our results suggested that gain-framed health messages are more effective to motivate self-care behaviors, whereas loss-framed health messages are more effective to communicate the risk of contagion.

Contrary to our expectation, gain-framed messages were more effective to motivate both self-care behaviors (i.e., not only hand washing, but also staying home). This result may have occurred because self-care behaviors have been previously associated with the avoidance of contagion with COVID-19, and avoiding contagion is a form of gain. In short in length messages (like the ones used in this study), it is easier to execute the frame (gain or loss), with which the behavior has been previously associated. In a similar vein, since the spread of COVID-19 is already associated with negative consequences in public awareness, a loss-frame might be more effective to increase risk perception despite the short length of the message. Similar results were found previously in warning labels in the context of quit smoking (Goodall and Appiah, 2008; Nan et al., 2015).

In mass media and on social networks space is limited (e.g., the maximum length of a tweet is 280 characters); our study results thus suggest that messages designed in this kind of media to motivate self-care behaviors to avoid the spread of COVID-19 should use a gain-frame structure. Conversely, if the target is to improve risk perception, a loss-frame message will be more effective.

Health content messages had a greater impact on the main variables studied, however, participants tended to assess other people as more influenced by economic content than themselves. This result may indicate the beginning of a growing concern about the economic situation of the country, although reflected for now in the economic situation of other people (McKee and Stuckler, 2020; Singer and Plant, 2020; Tankersley, 2020). Because the participants of the present study belonged to a medium-high socioeconomic level, they have not yet experienced the negative economic consequences of the lockdown, but they are aware of the economic difficulties of other citizens through the media, social networks, and direct experience, especially in a country with a fragile economy. Notice that this study was conducted at a moment when the lockdown was the only widespread measure against the spread of the virus in the country and other measures that have proven successful had not been implemented (e.g. mask wearing), which needs to be factored in when judging the behavior of others (complying with the stay at home orders). This mismatch between preferences reported by participants and those attributed to others opens the way for potential interventions based on social norms feedback (Dempsey et al., 2018): since larger misperceptions tend to be associated with a greater likelihood of engaging in negative behaviors (e.g., in this context, not complying with self-isolation recommendations), message delivery based on adequately communicating true rates of observance of recommendations could be a promising strategy.

Governments are going to great lengths to communicate and persuade the general population of the best measures to prevent COVID-19 spread. Many of these messages are disseminated through mass media and social networks, where time and space are limited. The results of the present study suggest that gain-framed health messages are more effective to motivate people to engage in self-care behaviors, additionally, these increase the attention, perceived importance, consequences expressions, and perceived effectiveness (i.e., increase perceived message strength). Conversely, loss-framed health messages improve risk perception. This is especially important in some populations where risk perception is usually low (e.g., adolescents), and because recent studies have found that risk perception is significantly correlated with reported adoption of self-care behaviors to prevent COVID-19 contagion (Dryhurst et al., 2020; Lohiniva et al., 2020). These results may be used to develop more effective messages by policy makers.

The present study has several limitations. First, only self-report measures were evaluated, we do not have behavioral measures (e.g., behavior frequency) that allow us to corroborate the results. However, although several models identified that intention is not sufficient to adopt a new behavior, intention is a necessary step to adopt it (Prochaska, 2008; Schwarzer, 2008). Second, our results are not necessarily applicable to countries, regions or communities with different socioeconomic or cultural conditions. It is possible that high-income or very low-income countries or regions may have different responses to message framing and content. These results are then more likely to generalize to middle-income countries, in particular middle-class urban settings. Lastly, in a rapidly changing situation, our results offer only a snapshot of a set of concerns that evolve as the pandemic changes.

## Data Availability Statement

The preregistration, hypotheses, analysis plan, materials, raw data, and scripts for analysis are available online at the Open Science Framework (https://osf.io/mxa3q/).

## Ethics Statement

The studies involving human participants were reviewed and approved by Institutional Review Board of the Universidad de los Andes (approval #1169/2020). The participants provided their written informed consent to participate in this study.

## Author Contributions

CG and WJ-L are responsible for the original design of the experiments, stimuli design, data analysis, and writing of the manuscript. JU-R contributed to materials design, conducted the experiments, and contributed substantially to data analysis and drafting of the manuscript. All authors approved the final version of the manuscript for submission.

## Conflict of Interest

The authors declare that the research was conducted in the absence of any commercial or financial relationships that could be construed as a potential conflict of interest.

## Footnotes

1 https://osf.io/mxa3q/

## Supplementary Material

The Supplementary Materials are available at the Open Science Framework repository: https://osf.io/mxa3q/
